# Vagueness and volume: Testing the perception of depth in images with linear, sharp, or blurred contours

**DOI:** 10.1167/jov.24.4.12

**Published:** 2024-04-16

**Authors:** Jeroen F. H. J. Stumpel, Robert Volcic, Maarten W. A. Wijntjes

**Affiliations:** 1 Art History, Utrecht University, Utrecht, the Netherlands; 2 Perceptual Intelligence Lab, Industrial Design Engineering, Delft University of Technology, Delft, the Netherlands; 3 Division of Science, New York University Abu Dhabi, Abu Dhabi, United Arab Emirates; 4 Center for Artificial Intelligence and Robotics, New York University Abu Dhabi, Abu Dhabi, United Arab Emirates; 5 Center for Brain and Health, New York University Abu Dhabi, Abu Dhabi, United Arab Emirates

**Keywords:** contour, art history, depth perception, shape perception, vision and depiction

## Abstract

In European painting, a transition took place where artists started to consciously introduce blurred or soft contours in their works. There may have been several reasons for this. One suggestion in art historical literature is that this may have been done to create a stronger sense of volume in the depicted figures or objects. Here we describe four experiments in which we tried to test whether soft or blurred contours do indeed enhance a sense volume or depth. In the first three experiments, we found that, for both paintings and abstract shapes, three dimensionality was actually decreased instead of increased for blurred (and line) contours, in comparison with sharp contours. In the last experiment, we controlled for the position of the blur (on the lit or dark side) and found that blur on the lit side evoked a stronger impression of three dimensionality. Overall, the experiments robustly show that an art historical conjecture that a blurred contour increases three dimensionality is not granted. Because the blurred contours can be found in many established art works such as from Leonardo and Vermeer, there must be other rationales behind this use than the creation of a stronger sense of volume or depth.

## Introduction

This study concerns the perceptual effect of contour quality on perceived three-dimensional (3D) shape. The motivation stems from the frequent mitigation of contours in the history of European painting, and a proposed possible relation with the wish to create a stronger scene of plasticity. Hence, we approach this problem from the two directions of vision and depiction and will start by reviewing the latter.

### Depiction

In European painting, “illusionism” was one of the dominant aims since the 15th century. Some means to achieve it have been well studied and described, for example, the application of perspective, or of shadows and shading. The actual handling of paint as a way to create certain optical effects has been studied much less. Several older treatises discuss the blurring of the contours or edges of painted objects, which is the topic of this paper.

**Figure 1.**
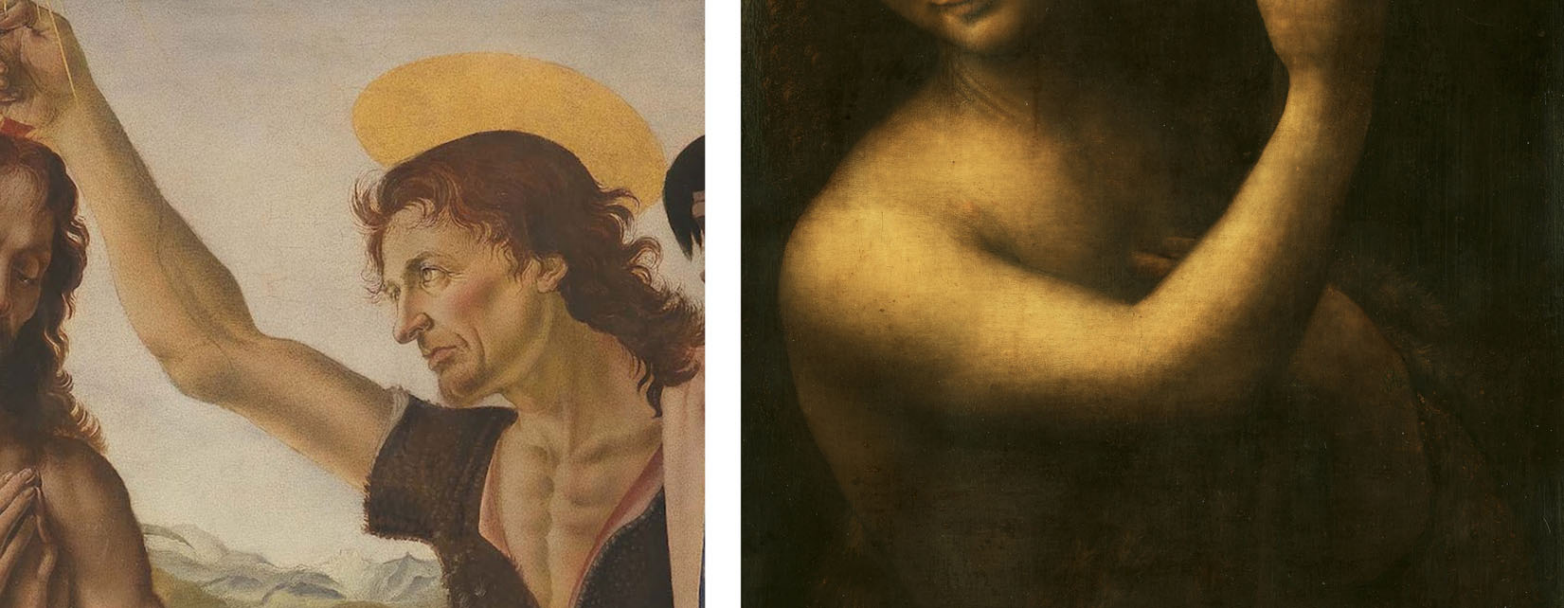
Left, a detail of Verrocchio’s St. John the Baptist, with a clear contour. Right, Verrocchio’s student, Leonardo Da Vinci depicts a similar motif, St. John the Baptist, but with clear sfumato contours.

A very early source is Leonardo, who spoke against the use of visible linear contours around the edges of objects in pictures, because they are generally absent in the natural world (Da Vinci, 1651). That Leonardo felt the need to argue this point because in contemporary Italian workshops the use of tempera paint had been dominant, and quite often indeed artists would show a kind of black contour. Within the confines of such a contour, colors were applied. The method might be compared with modern color books, where the line drawing is the dominant feature as well, and colors are applied later within these pre-established confines. Such procedures can be seen in many Italian paintings of the fourteenth and fifteenth century. Witness, for instance, this arm of St John the Baptist, painted by Leonardo's master Verrocchio (Figure 1, left).

Leonardo belonged to the first Florentine generation that fully switched to the use of oil paints on panel. This change of technique was due to the impact made by the integrated oil technique of painters from the low countries, which among other things showed no such linear contours. Oil paint has certain affordances that are lacking in egg-tempera paints (see Dunkerton, Foister, Gordon, & Penny, 1991, p. 152–205). One of these is the fact that the slow drying of such paints, combined with its transparency, allows for soft transitions and blending. This possibility, fully exploited already by the Early Netherlandish Masters, is the basis of what in Italian came to be called *unione*, a unifying softness of transitions between different colors and tones within the boundaries of objects. Leonardo wrote about a “smoky” procedure: “Make sure that your lights and shadows melt together without lines, like smoke.” But this same potential could be used for the softening of the contours themselves. We may read in the work of Daniele Barbaro in 1556, the advice to render “the contour soft and smokey, to include that which you cannot really see” (see Barbaro, 1556, p. 188). He apparently meant the continuation of the object beyond or behind the visual edge, or, in other words, an enhancement of a sense of volume. The contours or outer rims of different objects within a painting were not blurred in Early Netherlandish paining, and Leonardo was perhaps the first painter to expand on this possibility. We see the practice demonstrated for instance in an arm of St. John, painted by Leonardo decades after Verrocchio's example (Figure 1, right). In fact, the term *sfumato* (“smoked”) was later even tied to the blurred contour specifically: witness its definition in a famous 18th century dictionary of art and art terms (see Watelet & Levesque, 1792, p. 739): “Sfumato. It consists of a way of painting that is extremely soft, and leaves a kind of incertitude about where the contour terminates.”

Applying a blurred contour gained ground during the 16th century, both north and south of the Alps. The concept was later referred to, it seems, in treatises of Netherlandish painters, from Van Mander in the late 16th century, to Willem Goeree in the 17th century. Van Mander (1604/1973) criticized painters of earlier generations for being, what he called *“cantigh op den dag,”* that is, having sharp edges on the lit side of objects (the “day” side, as opposed to the shadow side). Authors supported the idea of a softness in the contours, to achieve a rounding of figures or members, but their statements are far from equivocal. Franciscus Junius in his “De Schilder-konst der Oude” (Junius, 1641), commenting on the practice of painters in Antiquity, wrote that painters “should also take great care with the contours [...] the compass or outline of the figures [should] be drawn with such nicety and such unfettered sweetness that the beholders think they see in it [...] what is invisible [...] that not only what lies behind it seems believable, but that it also appears to show what lies hidden there.”

**Figure 2.**
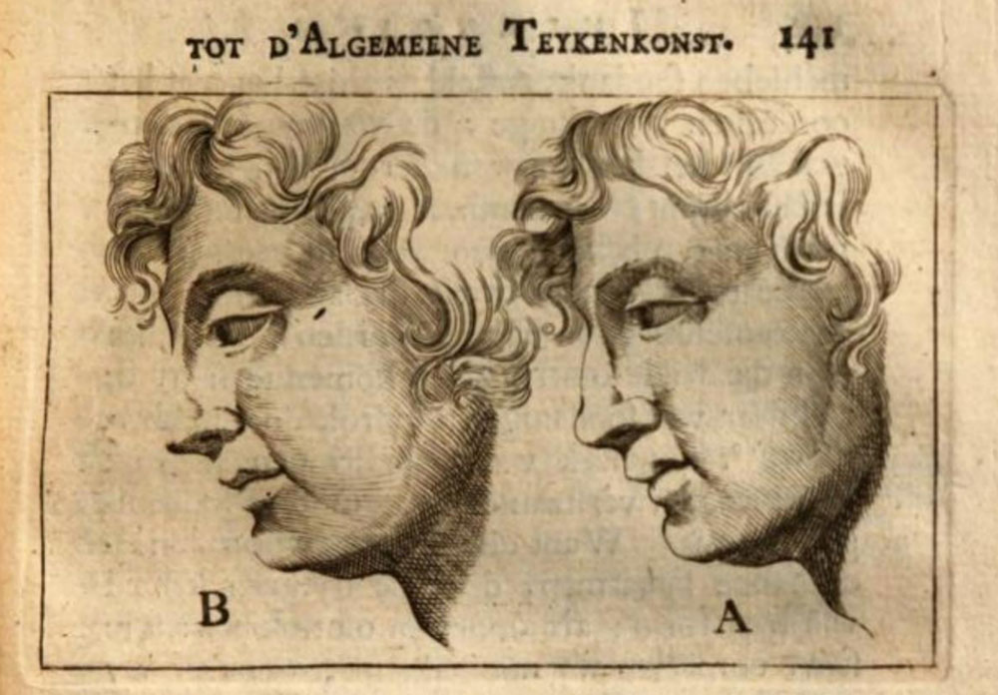
Illustration from Willem Goeree’s treatise.

This notion recurs elsewhere in Dutch art theory, up to Willem Goeree, who (like Leonardo much earlier) argued that in nature there are no “trecken” (Goeree, 1668), that is, no contours created by lines. Even when discussing the art of drawing, rather than painting, Van Mander for instance argued that the lines should be weak on the lit side of objects (the *dag* or day side), while they could be heavier on the shadow side. Later, Willem Goeree, again writing about drawing, realized that in this art one has to work with lines of course, but even so it could be better to locally omit them altogether sometimes, especially in those parts of objects that receive full (day-) light. The effect he demonstrated in two different drawings, one with a continuous contour, and another with the contour omitted here and there as shown in Figure 2. This demonstration was about drawing rather than painting, but the phenomenon is akin to painting techniques where contours were purposely made vague by locally blending and smudging the paint. If the texts on art may not always be that clear about the practice of locally avoiding sharp contours, paintings of the 16th and 17th centuries do show the phenomenon often enough. In a meticulous study, van Eikema Hommes (2005) has shown a variety of techniques with which a group of 17th-century painters did indeed establish locally blurred outlines in their pictures. She plausibly argues that such practices could be applied “to ensure that the figures do not appear to cease at their painted edges. In a painting it is precisely this suggestion that gives rise to the impression of depth and lends volume to the figures.” It might be argued that such blending does not actually occur in nature, just like its opposite, a distinct linear contour around objects. Given the many examples of artists using contour vagueness, we considered it worthwhile to try and obtain quantitative data about its supposed perceptual effects. We somehow expected that the instructions and practice of painters of the past about absent and/or blurred contours would be vindicated by experiments. Although there can be various perceptual effects of contour blur, we concentrated on the impression of depth and volume, as suggested by van Eikema Hommes (2005). Thus, we will continue with a review about the relation between blur, contours, and depth perception.

### Vision

Although 3D shape and the curvature of the occluding contour (Koenderink & Van Doorn, 1982) or terminating contour (Koenderink, 1984) are mathematically well-defined, artistic practice not always complies with the contour ending rule (Koenderink, 1984) and seems relatively ignorant toward saddle shapes (Koenderink, 2020). Yet, these studies concern the curvature of the contour, and not its style, that is, whether it is sharp, blurred, or outlined.

Although the influence of contour blur on depth perception has not been studied previously, the influence of blur per se did receive attention (Mather, 1996), demonstrated that sharp regions are perceived to have different depths than blurred regions. Although the blur itself does not contain information about the depth order, the contour blur does: when a contour is sharp, the blurred region is perceived to be further; when a contour is blurred, the blurred region is perceived closer than the sharper region (Marshall, Burbeck, Ariely, Rolland, & Martin, 1996). Interestingly, the contour blur needs to be relatively extreme to let the sharp region appear in the background, as was subsequently found (Mather & Smith, 2002). An inherent side effect of blurring is a lowering in contrast, which could also be used as a depth cue (O’Shea, Govan, & Sekuler, 1997) showed that contrast indeed strengthens the perceived depth difference, but when corrected for contrast, blur still affects depth perception. The role of defocus blur seems to complement information provided by binocular disparity although whether it a quantitative depth cue as claimed by Held, Cooper, & Banks (2012) is debated (Vishwanath, 2012).

What is important to note is that these studies refer to the depth separation between foreground and background, that is, the 3D spatial configuration. Our study aims to understand the effect of the contour on the three dimensional *shape*, which generally relies on a different set of depth cues like shading and texture (Todd, 2004). Whether contour blur affects 3D shape is currently unknown. A possible reason this has not been studied could be due to the absence of a physics rationale: optically, there seems no reason to suppose a relation between contour blur and shape. Many studies on 3D space and shape have been motivated by the geometry of projection and physics of light and reflection. Here instead, we find inspiration from an *artistic* conjecture that there is a relation between the contour style and shape. The visual system may use “alternative physics” (Cavanagh, 2005) and, moreover, may have acquired a new set or rules and conventions based on prolonged visual exposure to an ever changing visual environment. Therefore, artistic practice seems a relevant, complementary starting point next to the conventional psychophysics starting point, namely, the laws of physics.

In the experiments reported here, we empirically addressed the question of contour style on depth and volume. We started with the basic distinction between sharp and burred contours in the first experiment but included the “line” contour in subsequent experiments as this third contour style is clearly present in art history and discussed by Goeree (1668). Finally, we studied the subtlety of the position of blur, as proposed by Van Mander (1604/1973).

To operationalize our research question, we made use of (digital fragments of) paintings that in their original format possessed a sharp contour and compared these to artificially blurred versions (Experiment 1), originally possessed a blurred contour and compared these to artificially sharpened and outlined versions (Experiment 2), used synthetic stimuli that we rendered ourselves with blurred, sharp, and outlined contours (Experiment 3) and synthetic stimuli with contours that changed style depending on the body shadow (Experiment 4).

## Experiment 1

In the first experiment, we aimed to test the contour hypothesis by comparing original sharp contour depictions with manipulated blurred contours.

### Methods

#### Stimuli

**Figure 3.**
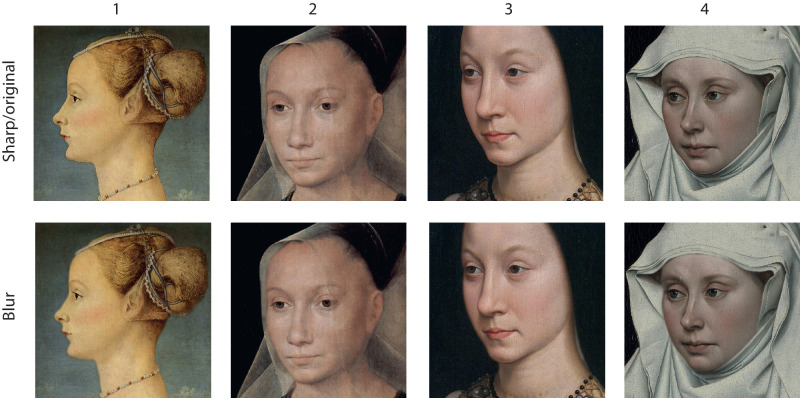
Overview of stimuli used in the first experiment. (1) Piero del Pollaiuolo *Portrait of a Young Woman* (c. 1471) Museo Poldi Pezzoli. (2) Hans Memling *Sibylla Sambetha* (1480) Old St. John’s Hospital. (3) Hans Memling *Portrait of Maria Portinari* (c. 1471) Metropolitan Museum of Art. (4) Robert Campin *Portrait of a Woman*(c. 1435.), National Gallery London.

We selected four painting fragments containing a female face that was depicted with a sharp contour. Because we were mainly interested in the existence of an effect per se, we did not parametrically vary the blur strength, but rather choose single blur values. We created blurry contours by using the blur tool in Adobe Photoshop 24.1.1 and set the size on 1/16th of the width of the image (800 pixels). We moved the blurring tool cursor along the contour until the effect was clearly visible. The original and altered images can be seen in Figure 3.

#### Participants

We recruited 60 participants on the online platform Prolific. We asked for participants to have a minimal education degree of bachelor and that they were fluent in English. The participants who joined the study were on average (standard deviation) 37 ± 12 years old. Thirty-seven participants identified as female, and 23 as male. The majority of the participant (41) came from the UK, others came from Germany, Ireland, Netherlands, Sweden, and the United States. The experiment was approved by the Human Research Ethics Committee of the Delft University of Technology and was conducted in agreement with the Declaration of Helsinki.

#### Procedure

Participants received the following instruction:

You will see pairs of images that look similar but are a little bit manipulated. You have to judge which object (faces in this case) seem to have more volume, more depth, and appears more three dimensional. We hope that you won’t try to “reason” too much but rather trust your visual instinct in making the judgments.

Each pair was repeated 3 times (although they were not presented in successive order) resulting in a total of 12 trials per participant. For each stimulus pair, we collected 180 judgments (60 participants times three repetitions). There was no time restriction. We logged response times and for each session we computed the median response time for each participant, as the median is less sensitive to outliers. The average response time over all median response times was approximately 3.2 seconds.

**Figure 4.**
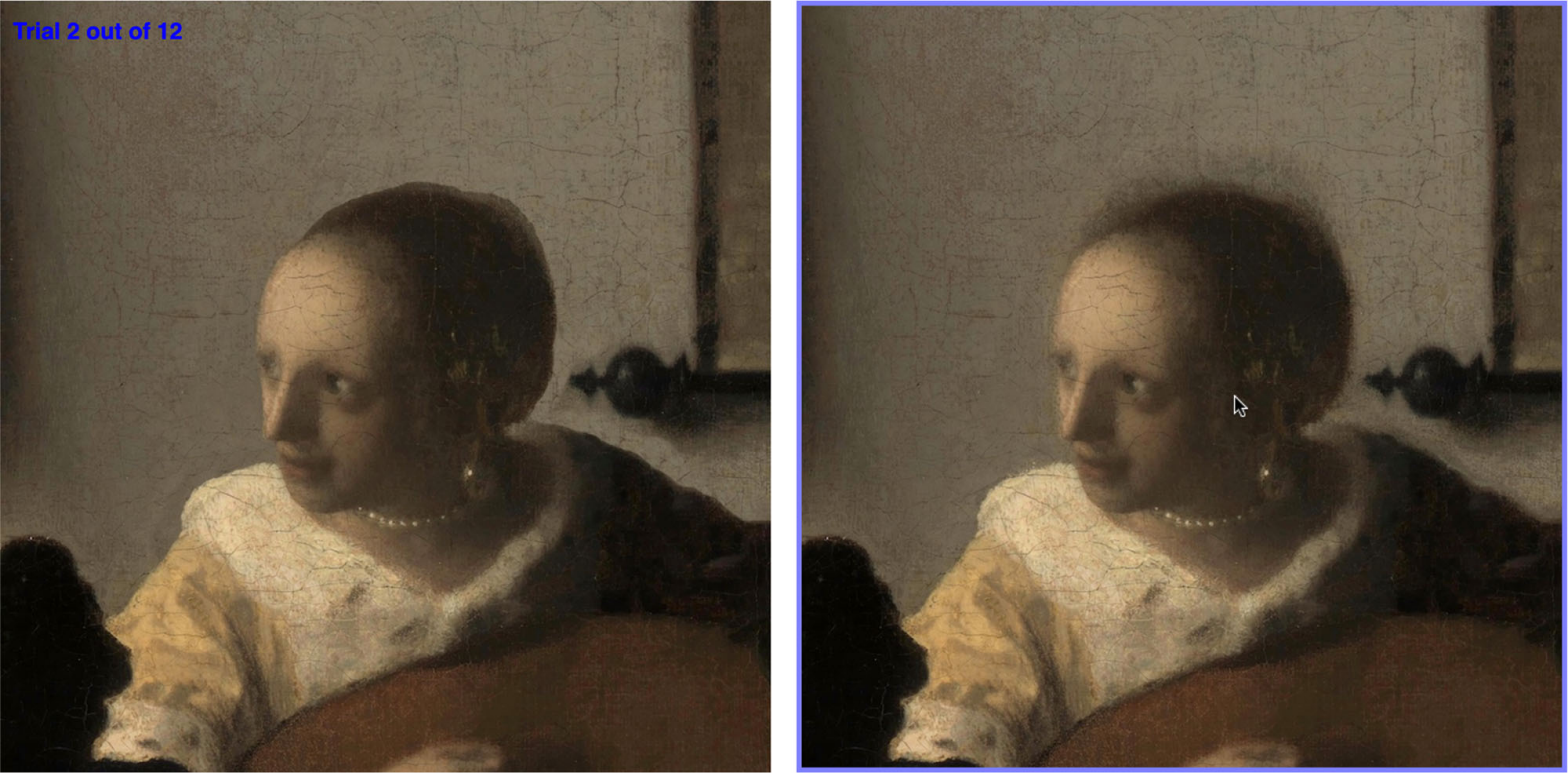
Screenshot of the (online) experiment. Participants clicked on the image to indicate their response.

Images were each displayed at 600 pixels width with 20 pixels white space between them. After a participant selected an image by clicking, the next trial proceeded automatically. A full screen version of the website is shown in Figure 4. As can be seen, the images were positioned side by side on a white background. A purple outline was shown around the image over which the mouse hovered.

#### Data analysis

Participants provided a binary response for each stimulus pair to indicate which of the two images (sharp vs blurred contours) appeared more 3D. These responses were fitted with a binomial generalized logistic regression mixed-effect model to take into account the nature of the responses and the repeated measures design of the experiment. This model provided separate estimates for each stimulus about the probability of choosing the sharp contour as the more 3D style. A second model was fitted to obtain a single probability estimate for all stimuli together. For each estimate, we performed parametric bootstrapping to obtain the 95% confidence intervals. For statistical inferences we assessed the overlap of the 95% confidence intervals with the chance level of 0.5.

### Results

**Figure 5.**
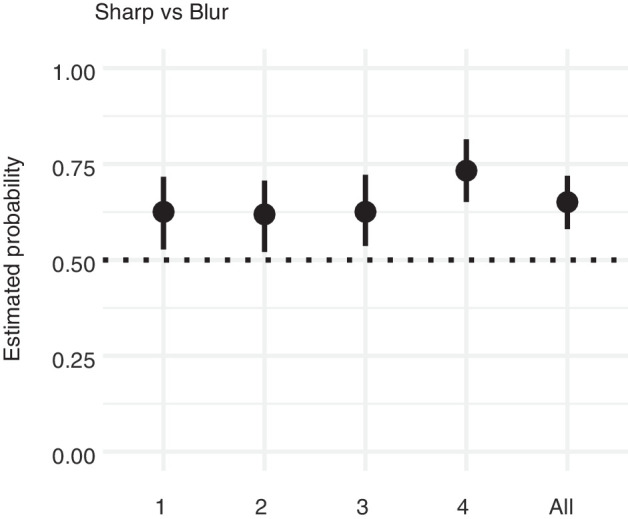
Results of Experiment 1. Proportion of choices favoring the sharp contour over the blurred contour for each stimulus (1, 2, 3, and 4) and for all stimuli together (all). Error bars represent the bootstrapped 95% confidence intervals. The horizontal dotted line represents chance level.

The results are presented in Figure 5. As can be seen, in all pictures the sharp (original) contour elicited the highest sense of three dimensionality. Although for the first three stimuli approximately 60% of the judgments favored the sharp contour, the last stimulus attracted even more than 70% of the judgments in favor of the sharp contour.

To assess the overall effect, we pooled all responses together (Figure 5, rightmost data point), which confirmed that participants judged the faces surrounded by sharp contours as having more volume and depth than those surrounded by a blurred contour.

### Discussion

The results clearly point in the direction opposite to our expectation. Instead of becoming more three dimensional, faces seem to loose depth when their contour is artificially blurred. Before discussing possible explanations and implications, we wanted to further test the robustness of this result. Because we artificially generated blurred contours in this first experiment, we wanted to conduct a follow up experiment where we used blurred contours as original material and create artificially sharp contours.

## Experiment 2

In the second experiment, we wanted to test the robustness of the results found in Experiment 1. We also wanted to add a third contour condition: the outline. As source material we needed faces with blurred contours and decided to choose four paintings of a true contour master: Johannes Vermeer (1632–1675).

### Methods

#### Stimuli

**Figure 6.**
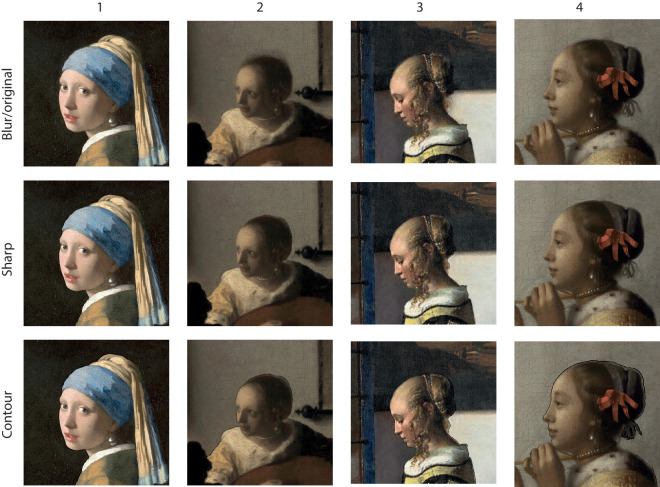
Overview of stimuli used in Experiment 2. All portraits are fragments from paintings by Johannes Vermeer. (1) *Girl with a Pearl Earring* (1665), Mauritshuis, The Hague. (2) *Woman with a Lute* (c. 1662–64), Metropolitan Museum of Art, New York. (3) *Girl Reading a Letter at an Open Window* (1657), Gemäldegalerie Alte Meister, Dresden. (4) *Woman with a Pearl Necklace* (c. 1662–64) Gemäldegalerie, Berlin.

We edited the contours of the faces in two ways. To increase the sharpness, we hand-segmented the face out of its background, using a contour path that excluded the blurry transition. Then we deleted the left over blurry region by using the stamp tool, overriding it with patches of the background region. Then we pasted the segmented face back. For the contour version, we drew a black contour over the blurry region of the contour. The original and altered images can be seen in Figure 6.

#### Participants

A total of 80 participants were recruited on Prolific; none of them had participated in Experiment 1. According to the demographic data provided by Prolific, the participants were on average (standard deviation) 39 ± 14 years old. The majority of the participants live in the UK (57); the remainder were distributed across Germany, Ireland, the Netherlands, Norway, Sweden, and the United States. A total of 46 identified as female and 34 as male. Data of two participants were lost due to server problems, thus totalling the number of actual data to 78.

#### Procedure

The procedure was largely similar to Experiment 1 with the exception that we did not have trial repetitions. However, because we now have an additional third condition (line contour) resulting in three pairs per painting, the total amount of trials still amounted to 12 trials per participant. The average response time over all participants was approximately 3.4 seconds.

#### Data analysis

The data analysis was the same as in Experiment 1 with the main difference that we compared three instead of two conditions. Participants provided a binary response for each stimulus pair to indicate which of the two images (sharp vs. blurred contours, blurred vs. line contours, sharp vs line contours) appeared more 3D. The responses of each comparison were fitted with a separate binomial generalized logistic regression mixed-effect model to take into account the nature of the responses and the repeated measures design of the experiment. These models provided separate estimates for each stimulus about the probability of choosing the sharp contour for the sharp vs blurred contours comparison, the blurred contour for the blurred versus line contours comparison, and the sharp contour for the sharp vs line contours comparison as the more three dimensional style. A second set of models was fitted to obtain single probability estimates for each comparison. For each estimate, we performed parametric bootstrapping to obtain the 95% confidence intervals. For statistical inferences we assessed the overlap of the 95% confidence intervals with the chance level of 0.5.

### Results

**Figure 7.**
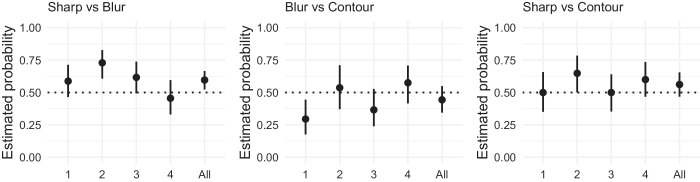
Results of Experiment 2. (Left) Proportion of choices favoring the sharp contour over the blurred contour. (Middle) Proportion of choices favoring the blurred contour over the line contour. (Right) Proportion of choices favoring the sharp contour over the line contour. Each panel represents the proportion of choices for each stimulus (1, 2, 3, and 4) and for all stimuli together (all). Error bars represent the bootstrapped 95% confidence intervals. The horizontal dotted line represents chance level.

The results are presented in Figure 7. For the sharp versus blurred contour comparison, the faces with the sharp contour were chosen as having more depth than the ones with the blurred contour. This effect was strongest for painting 2 with more than 70% of judgments in favor of the sharp contour. Paintings 1 and 3 elicited results of comparable magnitude (approximately 60%) as paintings 1, 2, and 3 from Experiment 1. The overall effect deviated from chance level replicating the finding from Experiment 1.

For the blurred versus line contour comparison, there was an above chance preference for the line contour eliciting more depth than the blurred contour only for painting 1. Overall, participants did not show any preference for either of the contour styles.

For the sharp versus line contour comparison, there was an above-chance preference for the sharp contour eliciting more depth than the line contour only for painting 2. The overall effect was not different from chance level.

### Discussion

Experiment 2 confirms the findings from Experiment 1: sharp contours make a shape (face) appear more 3D than blurred contours. When comparing the original blurred edges with line contours, we did not find an effect, except for the *Girl with Pearl Earring*. The explanation is likely trivial: because of the exceptionally dark background of this iconic image, the line contour becomes part of that background, which effectively transforms the contour into a sharp contour. Further proof for the equivalence of line and sharp contour for *Girl with the Pearl Earring* is found in the direct comparison that shows an equal amount of votes for the two conditions.

So far, the evidence indicates that sharp, instead of blurred contours enhance pictorial depth. Yet, our results are restricted to face depictions and only compared original with edited versions. In the next experiment, we wanted to investigate whether this finding generalizes to abstract 3D shapes.

## Experiment 3

By rendering four different 3D shapes and varying the contour in a more controlled fashion than the edited paintings, we aimed to investigate the generalizability of our previous findings.

### Methods

#### Stimuli

**Figure 8.**
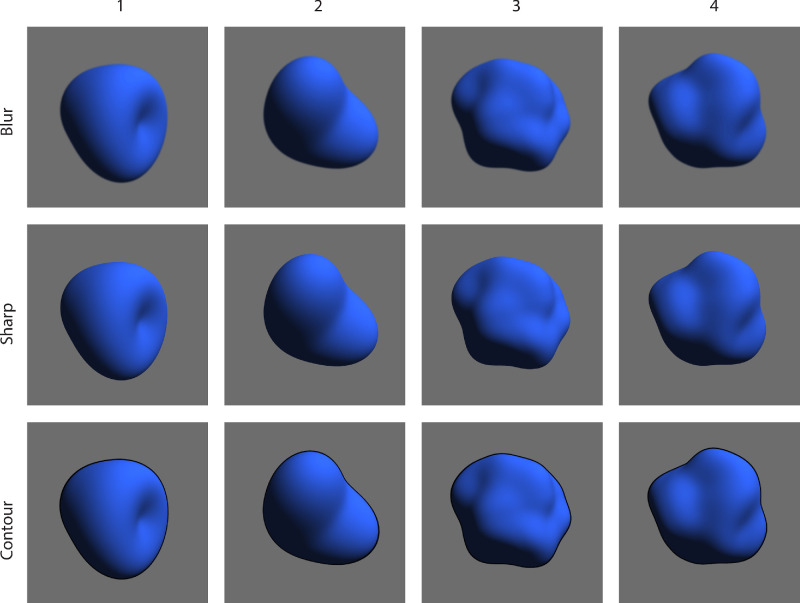
Overview of stimuli that consist of two parametric, simple shapes (1 and 2) and two randomly generated more complex shapes (3 and 4) rendered with a matte reflectance under identical illumination.

We rendered two simple and two more complex shapes (Figure 8). The first two are parametric shapes previously used in Nefs, Koenderink, and Kappers (2005), the complex shapes (number 3 and 4) were generated according to Derzsi and Volcic (2017). The sharp images were the originals and we blurred the edge by selecting a region around the contour defined by a scaling of 98% and 102% of the shape. This area was blurred with a Gaussian blur of 8 pixels wide. For the line contour, we filled the area between 100% and 102% scaling of the shape with a black color (including anti-aliasing to make it not too harsh).

#### Participants

We used Prolific to recruit 80 participants, none had participated in previous experiments of this study. According to the demographic data from Prolific, the participants were on average (standard deviation) 38 ± 12 years old. The majority of the participants live in the UK (59) and the remainder were distributed across Belgium, Germany, Ireland, the Netherlands, and the United States. A total of 47 identified as female and 33 as male. Data of four participants were lost due to server problems, thus totalling the number of actual data to 76.

#### Procedure

The procedure was the same as in Experiment 2. The average response time over all participants was approximately 3.7 seconds.

#### Data analysis

The data analysis was the same as in Experiment 2.

### Results

**Figure 9.**
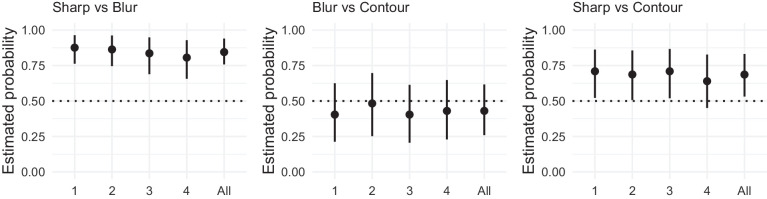
Results of Experiment 3. (Left) Proportion of choices favoring the sharp contour over the blurred contour. (Middle) Proportion of choices favoring the blurred contour over the line contour. (Right) Proportion of choices favoring the sharp contour over the line contour. Each panel represents the proportion of choices for each stimulus (1, 2, 3, and 4) and for all stimuli together (all). Error bars represent the bootstrapped 95% confidence intervals. The horizontal dotted line represents chance level.

The results are presented in Figure 9. Upon first impression, the results of the abstract stimuli seem to be more homogeneous than the results of the previous experiments with depicted faces. The differences between stimuli is much smaller between the abstract stimuli than the faces. Furthermore, the dominating effect of the sharp contour over the blurred contour became even stronger because more than 80% of the participants’ responses favored the sharp version compared with approximately 60% in Experiments 1 and 2.

There was no effect between the blurred contour and the line contour. Furthermore, the effect of sharp contours eliciting more depth than line contours was present, although it was weaker than the effect of sharp contours over blurred contours.

### Discussion

The results are completely in line with the previous two experiments that made use of paintings, while Experiment 3 concerned computer renderings of abstract shapes. To some extent, the results of all three experiments point towards a clear rejection of the hypothesis that blurred contours enhance the 3D appearance of shapes. Before trying to explain this in the general discussion, there is one remaining question for which we sought empirical insights. Although the historical accounts suggest blurring only contours on the lit side (the “day” side) of shapes, we have blurred the complete contours in all our foregoing experiments. This had partly practical reasons because there is much asymmetry between the light and dark sides of the depicted faces, but because we used abstract stimuli in Experiment 3, this barrier is no longer present.

## Experiment 4

To understand the influence of the blur placement, we adapted the stimulus set used in Experiment 3 by blurring only specific regions of the contours. Van Mander (1604/1973) seemingly rejects sharpness on the lit side, in favor of a certain level of blur on that part of the shape. We were also interested what would happen if we reverse this contour recipe: blurring the dark/shadowed side while keeping the lit part sharp. Hence, we compared two versions (blur on light and dark sides) with the sharp stimulus, which has trumped perceived depth in all previous experiments.

### Methods

#### Stimuli

**Figure 10.**
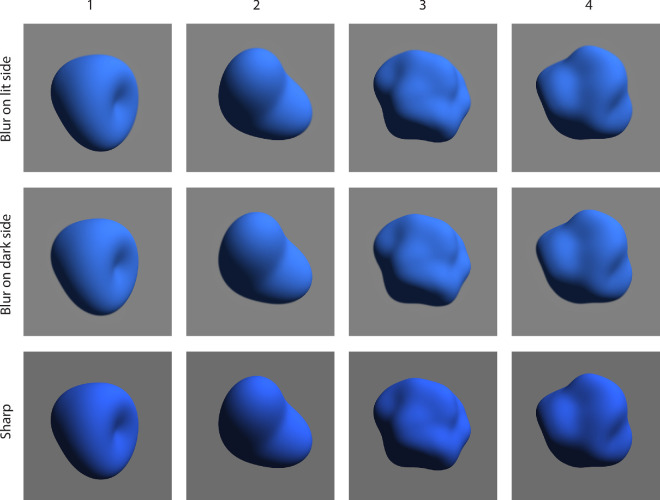
Overview of stimuli which are identical to those used in Experiment 4 except for the contours. Here, the contour blur was split into the lit and dark side.

In the photo editing software, we created two layers with the sharp stimulus on top and the blurred stimulus on the bottom. By erasing the edge with a large tool tip (80 pixels) with zero “hardness,” we created a controlled and smooth transition between sharp and blurred contour. We hand selected the dark and light side areas, which were complementary and together covered the complete outline. The images used as stimuli can be seen in Figure 10.

#### Participants

We used Prolific to recruit 80 participants, none had participated in previous experiments of this study. According to the demographic data from Prolific, the participants were on average (standard deviation) 39 ± 12 years old. The majority of the participants live in the UK (63) while the remainder were distributed across Belgium, Germany, Ireland, the Netherlands, Sweden, and the United States. A total of 37 identified as female, 42 as male, and 1 did not prefer to say (these are the only 3 gender options Prolific provides when asking the participants for demographic information). Data of seven participants were lost due to server problems, thus totaling the number of actual data to 73.

#### Procedure

The procedure was the same as in Experiments 2 and 3. The average response time over all participants was approximately 4.3 seconds.

### Data analysis

The data analysis was the same as in Experiments 2 and 3 with the exception that the analyzed comparisons were blur on lit side versus blur on dark side of the contour, sharp contour versus blur on lit side of the contour, and, sharp contour versus blur on dark side of the contour. The fitted models thus provided separate estimates about the probability of choosing the blur on lit side of the contour for the blur on lit side versus blur on dark side of the contour comparison, the sharp contour for the sharp contour versus blur on lit side of the contour comparison, and the sharp contour for the sharp contour versus blur on dark side of the contour comparison as the more 3D style.

### Results

**Figure 11.**
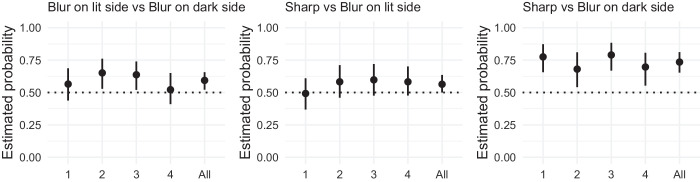
Results of Experiment 4. (Left) Proportion of choices favoring the blur on lit side contour over the blur on the dark contour. (Middle) Proportion of choices favoring the sharp contour over the blur on the lit side contour. (Right) Proportion of choices favoring the sharp contour over the blur on the dark side contour. Each panel represents the proportion of choices for each stimulus (1, 2, 3, and 4) and for all stimuli together (all). Error bars represent the bootstrapped 95% confidence intervals. The horizontal dotted line represents chance level.

The results are shown in Figure 11. For the blur on the lit side versus blur on the dark side of the contour comparison, stimuli 2 and 3 showed that participants chose more often the stimuli with the blur on the lit side as having more depth than the ones with the blur on the dark side. Even though the choices for stimuli 1 and 4 were essentially random, the overall results favor the blur on the lit side style.

For the sharp contour versus blur on the lit side of the contour comparison, there was a weak preference for the sharp contour style. In contrast, for the sharp contour versus blur on the dark side of the contour comparison, we found a clear preference for the sharp contour as the style eliciting more volume and depth.

### Discussion

This last experiment finally reveals some empirical evidence for the art historical hypothesis about contours. However, the effect clearly needs to be reframed in different terms. As Experiments 1 to 3 showed, sharp contours cause a shape to seem to be more 3D than blurred or outlined contours. What Experiment 4 shows, is that the effect of sharpness is particularly prominent in the dark, shadowed area of a 3D shape. This is a different framing than claiming that blur on the lit side increases apparent depth. Both claims are equivalent in this context, but emphasizing the effect of sharpness seems, given the results of all experiments, more appropriate.

## General discussion

We found a robust perceptual effect of contour blur on perceived volume that generalizes over paintings of faces and renderings of abstract shapes. However, the direction of the effect was opposite to our art historical hypothesis: a sharp edge may make a shape more voluminous.

One possible reason for this finding is that we have misinterpreted the art historical sources. While it has been supposed among art historians that the blurring is related to a more marked plasticity, (c.f. see Wetering, 1997; van Eikema Hommes, 2005), actual sources such as Leonardo, Van Mander, and Goeree primarily speak about their preference for a blurred contour, not necessarily their effect on volume.

There is no doubt that the depiction of contour has evolved from sharp and line contours towards a rather frequent use of more blurred contours. It can also be seen that soft contours are primarily discussed and used for body parts (although they sometimes may be seen in fabrics), but a quantitative annotation study would be needed to verify this. It makes one wonder about other perceptual effects that the painters were regularly aiming for. One effect that we observed informally is that a blurred contour affects the material qualities of the objects. A blurred contour could alter the object to appear softer or smoother. For example, the abstract rendered shapes in Figure 8 seem to change the overall appearance while only the contour is blurred. We found that many lab members thought the overall shape was blurred, and not only the outline, akin to the famous watercolor illusion (Pinna, Brelstaff, & Spillmann, 2001). Also the effect of our photo editing on the painted faces seem to suggest to some that the skin appeared less smooth when sharpening the contour. It may be worthwhile pursuing this question in future studies especially combined with a further questioning of the historical art sources on this point.

A motivation that is clearly documented is to “include that which you cannot really see” (Barbaro, 1556), “show what lies hidden there” (Junius, 1641). It could be that the intended effect is merely this, to not let the background come to a sudden halt but suggesting it continuous existence behind the occluding object. Thus, the contour could support the Gestalt law of good continuation.

As discussed in the Introduction, there is a strong relation between blur and depth, although not with respect to volume but rather to depth separation. Defocus blur is a significant depth cue and through contour blur observers decide what is foreground and background (Marshall et al., 1996). A contemporary photograph made with a large aperture and thus a shallow depth of field typically uses the convention to put the protagonist sharp in the foreground, and the scene/narrative in the blurry background. The painterly convention of using blurred contours contrasts a bit with this contemporary usage. Nevertheless, the blurred contours could indeed have be used as depth cues in our experiments. Although our instructions emphasized perceived depth of the face/object (“Which face is more 3D, has more depth/volume?”), it could well be that some participants have misinterpreted this and instead answered with respect to depth difference between foreground and background.

It should be noted that we used contemporary eyes to test an historical hypothesis. Nowadays, people have been exposed to innumerable photos with blurry backgrounds (and sometimes foregrounds), that follow optical laws of lenses and projections. This visual convention did not exist before the invention of the photo camera. Moreover, the sheer number of these images that humans are exposed to today is much higher than, say, 100 years ago, let alone during the time of Da Vinci or Vermeer. We may never know whether art work contemporaries perceived the depth differently, but we should definitely consider this possibility.

Although the initial motivation of this paper was to study a hypothesis of depiction in the domain of vision, the hypothesis was not supported by our findings, which implies we should reconsider the interpretation of the historical sources. However, our results imply that vision research may have important philological consequences for our interpretations of written art theoretical sources, as well as impact our views of the art works of the past.
